# A Potassium Based Fluorine Containing Bioactive Glass for Use as a Desensitizing Toothpaste

**DOI:** 10.3390/molecules26144327

**Published:** 2021-07-17

**Authors:** Melissa Tiskaya, David Gillam, Saroash Shahid, Robert Hill

**Affiliations:** Dental Physical Sciences Unit, Centre for Oral Bioengineering, Institute of Dentistry, Barts & The London School of Medicine and Dentistry, Queen Mary University of London, Floor 2 Francis Bancroft Building, Mile End Road, London E1 4NS, UK; d.g.gillam@qmul.ac.uk (D.G.); s.shahid@qmul.ac.uk (S.S.); r.hill@qmul.ac.uk (R.H.)

**Keywords:** bioactive glass, potassium, toothpaste, dissolution, remineralization, bioactivity

## Abstract

Potassium releasing bioactive glasses (BAGs) may offer improved relief for dentine hypersensitivity compared to conventional sodium containing BAGs by releasing K^+^ ions for nerve desensitization and occluding dentinal tubules to prevent fluid flow within dentinal tubules. Potassium oxide was substituted for sodium oxide on a molar basis in a fluoride containing BAG used in toothpastes for treating dentine hypersensitivity. The BAG powders were then immersed in an artificial saliva at pH 7 and tris buffer and the pH rise and ion release behavior were characterized by ICP-OES and ISE. The potassium and sodium containing BAGs were characterized by XRD, DSC, FTIR and NMR. Both BAGs presented amorphous diffraction patterns and the glass transition temperature of the potassium glass was higher than that of the sodium glass. The ^31^P MAS-NMR spectra indicated a peak at 2.7 ppm corresponding to apatite and a small peak at −103 ppm indicated crystallization to fluorapatite. Both BAGs dissolved and formed apatite at similar rates, although the dissolution of the potassium glass was slightly slower and it released less fluoride as a result of partial nanocrystallization to fluorapatite upon quenching. The potassium release from the potassium ions could potentially result in nerve deactivation when used in toothpastes.

## 1. Introduction

Bioactive glasses (BAGs) are attractive additives for remineralizing toothpastes for treating dentine hypersensitivity [1,2,3,4,5,6,7,8,9,10,11,12,13,14]. Dentine hypersensitivity is thought to occur as a result of fluid flow in open dentinal tubules that triggers nerves in the pulp chamber [15]. Toothpastes for treating dentine hypersensitivity work in two distinct ways [16]:

1. By occluding the open tubules thereby preventing fluid flow.
2. By providing K^+^ ions typically in the form of potassium nitrate that interfere with nerve excitation.

Bioactive glass toothpastes the typical compositions of which are given in Table 1 have been introduced to the market for treating dentine hypersensitivity. The first study on the ability of a bioactive glass to occlude dentinal tubules was published by Gillam et al. [1] using the 45S5 glass composition given in Table 1 that is now marketed under the trade NovaMin^®^. Subsequently, a high phosphate fluoride containing bioactive glass was developed by BioMin Technologies specifically for toothpastes.

Conventional bioactive glasses dissolve in physiological fluids releasing Ca^2+^ and orthophosphate (PO_4_^3−^) ions and form hydroxyapatite. They were originally developed for bone substitutes but have found application more recently in toothpastes where the glass is sold under the trade name NovaMin^®^ [17]. Brauer, Hill and coworkers developed high phosphate and fluoride containing bioactive glasses that release F^−^ ions in addition to Ca^2+^ and PO_4_^3−^ ions and form fluorapatite (FAp), the fluoride analog of the hydroxyapatite tooth mineral [18,19,20,21,22]. Due to their higher phosphate content these glasses form apatite much faster and in greater amounts than NovaMin [23,24]. In addition to forming apatite, bioactive glasses also raise the local pH, since the first step in the degradation of the glass involves ion exchange of H^+^ ions from the immersion solution with Ca^2+^ and Na^+^ ions in the glass structure.

Some researchers have found that toothpastes containing potassium salts have provided a reduction in clinical symptoms of dentine hypersensitivity, suggesting that potassium may cause nerve desensitization, relieving sensitivity symptoms [25,26,27]. However, the proposed mechanism responsible for this has not been clinically proven. Therefore, bioactive glasses where Na_2_O is replaced partially or completely by K_2_O are potentially attractive, since the glass particles could both occlude the dentinal tubules and deliver K^+^ ions to cause nerve deactivation. The fact that K^+^ ions originate from the glass could avoid the problem of K^+^ ions being rapidly cleared from the saliva by salivary flow. Release from a glass potentially results in a more constant K^+^ ion concentration as a function of time and make them more effective in treating dentine hypersensitivity. Physically locating the glass particles of appropriate size within the dentinal tubules should make them particularly effective.

Previous studies by Elgayar et al. [28] have shown that Na_2_O can be replaced by K_2_O with little if any change in the dissolution or apatite formation behavior of the glass and little if any change in the structure of the glass. However, these studies were performed with low phosphate content glasses containing no fluorine. 

This study explores the complete substitution of K_2_O for Na_2_O in the BioMinF toothpaste glass composition shown in Table 1, which contains a higher phosphate content than the NovaMin glass, plus it has a small amount fluorine within the glass structure. It will explore the influence of potassium substitution for soda on the structure of the glass, the dissolution properties including potassium release and ability to form apatite. The BioMinF glass will hereafter be referred to as Na-FBAG and the potassium version will be referred to as K-FBAG.

## 2. Materials and Methods

### 2.1. Glass Synthesis

Two bioactive glasses (Table 1) were prepared using the melt-quench route. In the case of the potassium containing glass, the soda was replaced by potassia on a molar basis. Mixtures of analytical grade SiO_2_ (Prince Minerals Ltd., Stoke-on-Trent, UK), P_2_O_5_, CaCO_3_, K_2_CO_3_, Na_2_CO_3_ and CaF_2_ (Sigma–Aldrich, Gillingham, UK) were melted in a platinum–rhodium crucible for 1 h at 1400 °C in an electric furnace (EHF 17/3, Lenton, Hope Valley, UK). A batch size of 200 g was used. After melting, the glasses were rapidly quenched into water to prevent crystallization and dried overnight in an 80 °C oven. After drying, the glass frit was ground using a vibratory mill (Gyro mill, Glen Creston, London, UK) for 7 min twice and sieved using a 38 μm mesh analytical sieve (Endecotts Ltd., London, UK). The amorphous structure of the glasses was confirmed by powder X-ray diffraction (XRD; X’Pert PRO, PANalytical, Cambridge, UK).

### 2.2. Characterization Techniques

A Malvern particle size analyzer (Mastersizer 3000, Malvern Instruments Limited, Malvern, UK) was used to determine the particle sizes of the bioactive glasses, which are presented in Table 2. The two particle size distributions were found to be very similar. Differential scanning calorimetry (DSC, Stanton Redcroft DSC1500, Rheometric Scientific, Epsom, UK) was used with matched pair platinum crucibles to determine the glass transition and peak crystallization (Tc) temperatures of the glasses. Fifty milligrams of fine (<38 μm) and frit (1–2 mm) glass powder was run against an alumina reference at a heating rate of 20 °C/min from 25 to 1000 °C. ^19^F and ^31^P solid state magic angle spinning-nuclear magnetic resonance (MAS NMR, Bruker 600 MHz, Coventry, UK) was performed on the glass powders before and after immersion and samples were packed in 2.5 mm rotors. The ^19^F NMR data were collected at a Larmor frequency of 565.686 MHz under spinning conditions of 22 kHz. A fluorine-free probe was used, making background subtraction unnecessary. The ^19^F chemical shift scale was referenced using the −120 ppm peak of 1 M NaF aqueous solution as a secondary reference against CFCl_3_. For ^31^P NMR, 85% H_3_PO_4_ was used as a reference and the chemical shifts were adjusted to 0 ppm. The resonance and spinning frequency used was 243 MHz and 12 kHz respectively. X-ray diffraction (XRD, Panalytical, X’Pert Pro, Malvern Panalytical Ltd., Malvern, UK) and the attenuated total reflectance-Fourier transform infrared spectroscopy instrument (ATR-FTIR, Perkin Elmer, Frontier, MA, USA) were used to characterize the crystallinity of the glass powders before immersion and precipitation of an apatite layer on the surface of the glass particles after immersion. The supernatant solutions were characterized using a pH meter (Seven2Go Pro pH meter, Mettler-Toledo Ltd., Leicester, UK) and fluoride concentrations was determined using an ion selective electrode (ISE, ELIT 003n, Nico2000 Ltd., London, UK). The concentrations of Si, Ca, K, Na and P in solution were measured using inductively coupled plasma-optical emission spectroscopy (ICP-OES, Thermo Fisher iCAP 7400 Duo, Waltham, MA, USA).

### 2.3. Immersion Media Preparation

Remineralizing artificial saliva (AS7) was prepared according to Ten Cate et al. [29]. AS7 was prepared by dissolving 0.4411 g of CaCl_2_·2H_2_O, 0.245 g of KH_2_PO_4_, 9.532 g of HEPES and 19.386 g of KCL (all Sigma-Aldrich, Gillingham, UK) in 1600 mL of deionized water. The pH was adjusted to 7 by adding 0.5 M KOH (Sigma-Aldrich, Gillingham, UK) and deionized water was then added to make up a total volume of 2000 mL. The solution was stored in a fridge at 37 °C prior to use. Tris buffer (TB) solution (0.062 mol L^−1^) was prepared by dissolving 15.090 g of tris(hydroxymethyl)aminomethane (Sigma Aldrich, Gillingham, UK) in 1600 mL of deionized water. The pH was adjusted to 7.30 by adding 1 M hydrochloric acid and the solution was made up to 2000 mL. TB was stored in an incubator at 37 °C prior to use. TB with no K^+^ or Na^+^ ions was used to study Na and K release since the AS7 solution already contains significant amounts of Na and K.

### 2.4. Immersion Study

A total of 75 mg of glass powder was immersed in 50 mL of the AS7 for 0, 3, 6 24, 72, 168, 336 and 772 h. After immersion the glass powder was filtered off using Whatman filter paper (particle retention 5–13 μm) and dried overnight for subsequent analysis by ATR-FTIR, DSC and MAS-NMR. The solutions resulting from the immersion were used for ion release measurements using ICP-OES and ISE and pH changes were also monitored.

## 3. Results

**Figure 1:**
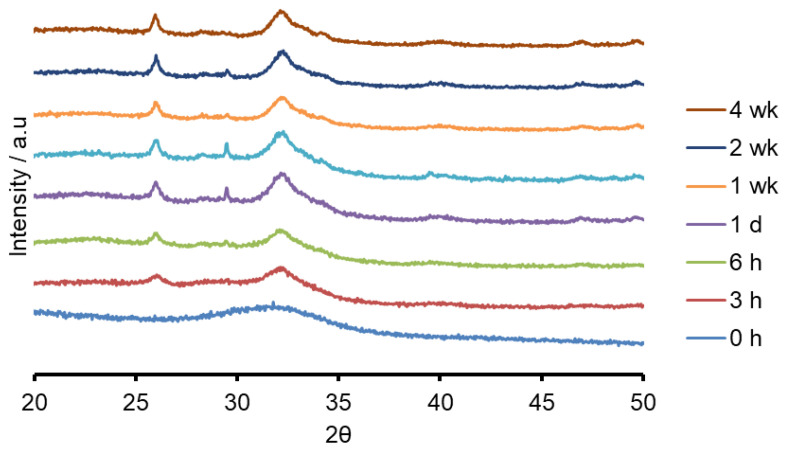
XRD patterns of Na-FBAG before and after immersion.

**Figure 2:**
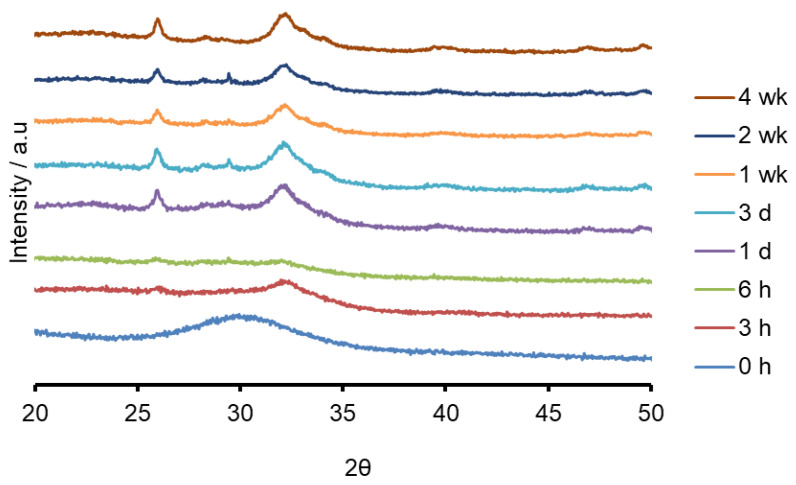
XRD patterns of K-FBAG before and after immersion.

**Figure 3:**
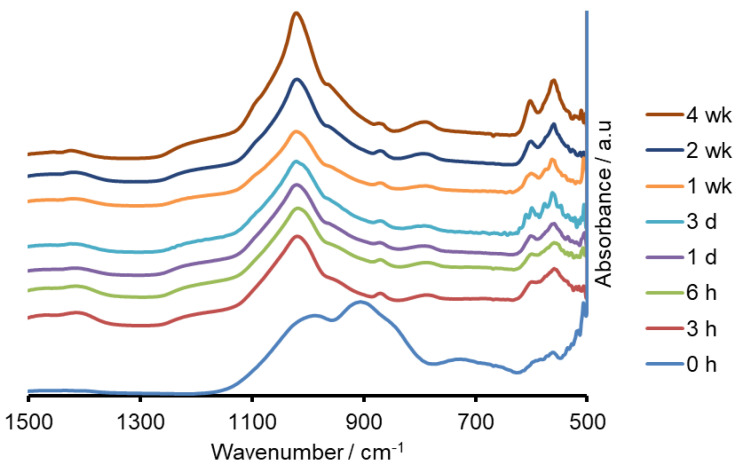
FTIR spectra of Na-FBAG before and after immersion.

**Figure 4:**
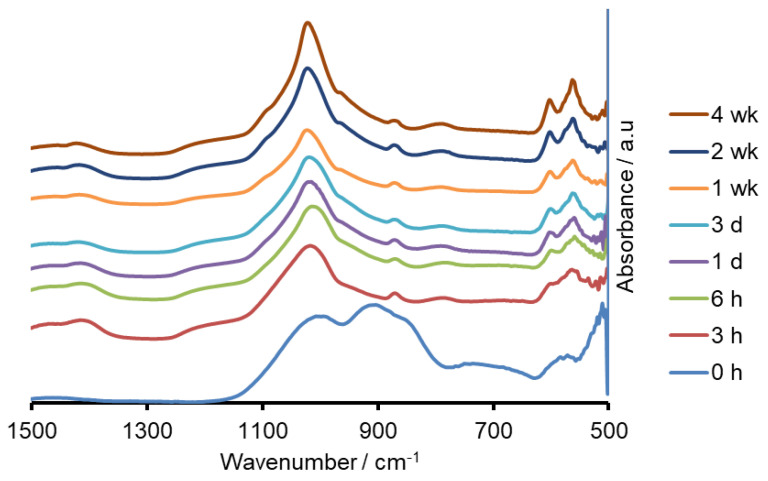
FTIR spectra of K-FBAG before and after immersion.

**Figure 5:**
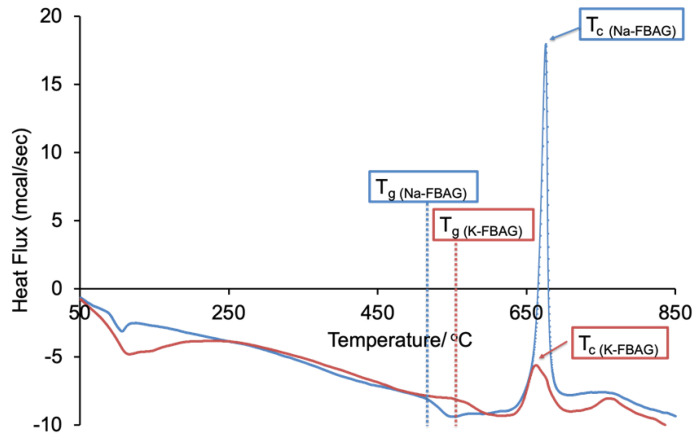
DSC traces of Na-FBAG (●) and K-FBAG (●).

Both the sodium containing (Na-FBAG) and potassium containing (K-FBAG) glasses were completely amorphous by XRD (Figure 1 and Figure 2). ATR-FTIR spectra of the Na-FBAG glass and K-FBAG glass are shown in Figure 3 and Figure 4. The spectra for the Na-FBAG and K-FBAG glasses show a broad peak at about 560 cm^−1^ but in the case of K-FBAG glass there was evidence of the superposition of weak bands at 560 and 600 cm^−1^ on top of the broad peak. In addition, both glasses exhibited broad bands at 860 cm^−1^ and 920 cm^−1^ corresponding to non-bridging oxygen vibrations in the glass structure and a broad peak at approximately 1000 cm^−1^. The DSC traces for both glasses are shown in Figure 5. Both glasses exhibited a clear glass transition temperature followed by a crystallization exotherm.

**Figure 6:**
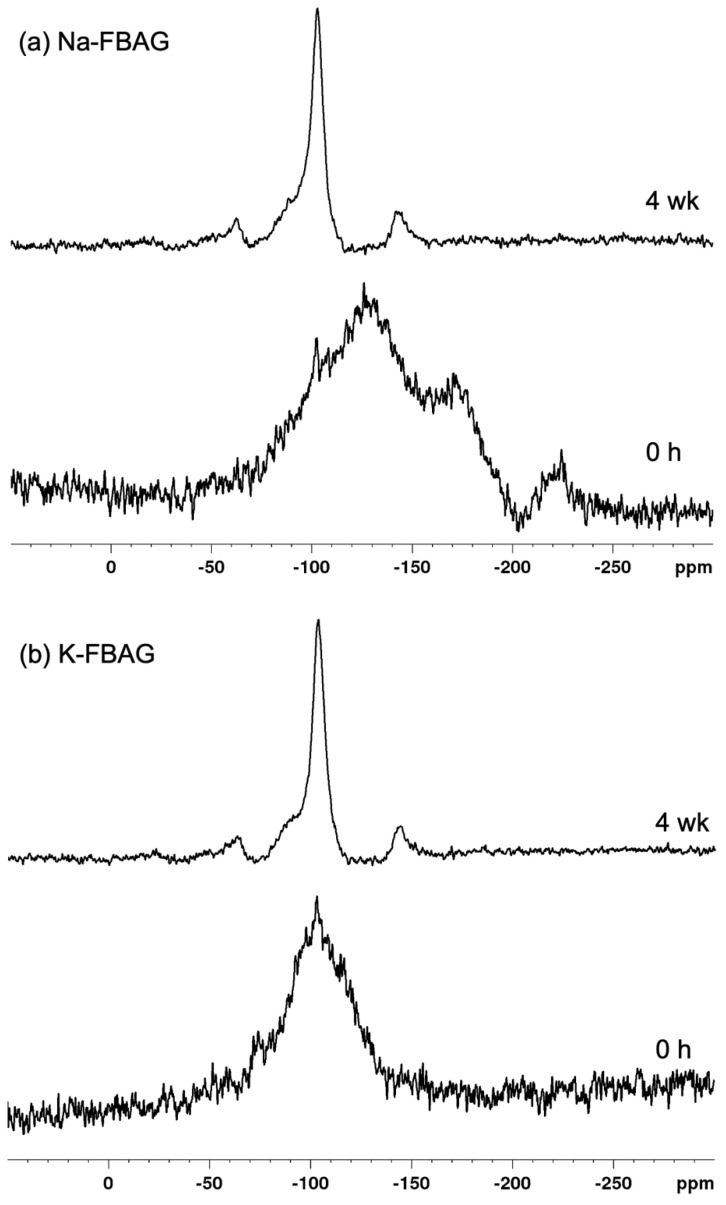
^19^F MAS-NMR spectra for (**a**) Na-FBAG and (**b**) K-FBAG.

**Figure 7:**
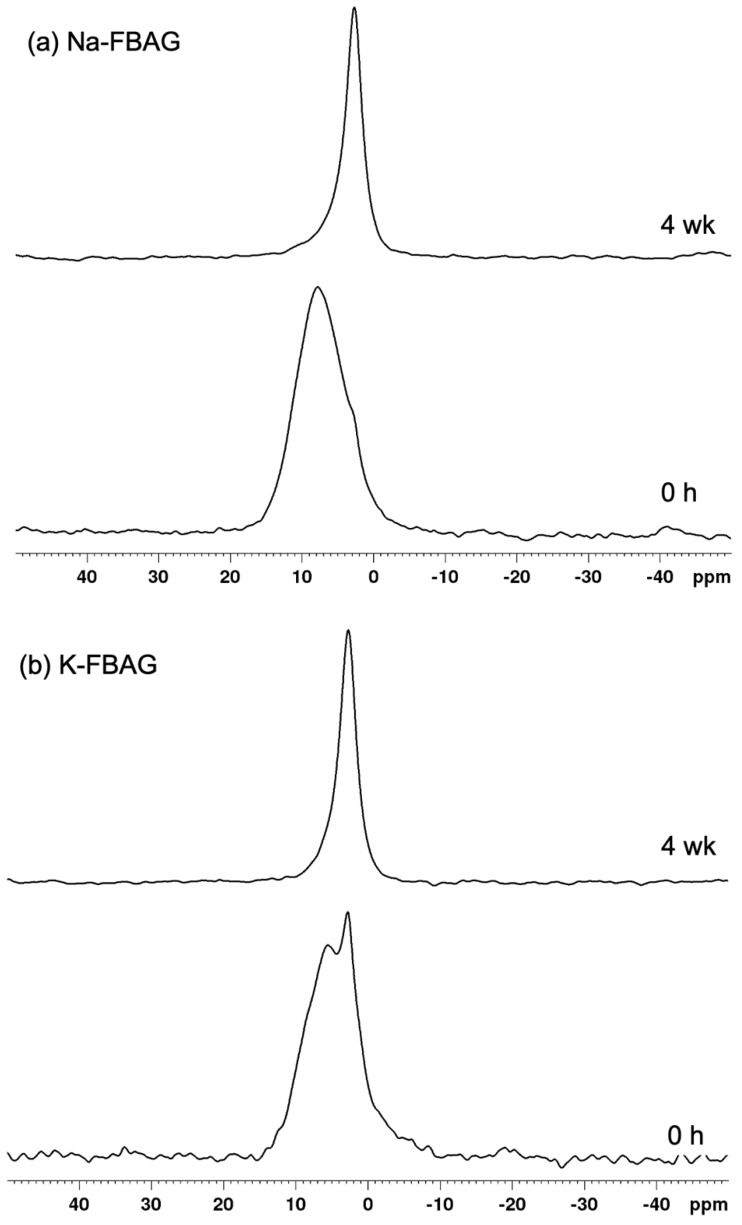
^31^P MAS-NMR spectra for (**a**) Na-FBAG and (**b**) K-FBAG.

The ^19^F spectra of Na-FBAG and K-FBAG are shown in Figure 6. In the case of Na-FBAG, the spectrum exhibited broad peaks at −130 ppm, −170 ppm and −220 ppm before immersion. There was a small peak at -103 ppm, suggesting crystallization to FAp. In the case of K-FBAG, a broad peak symmetrical peak at about −100 ppm was present with a sharp spike at −103 ppm, which also corresponded to FAp. The ^31^P MAS-NMR spectra of both glasses are shown in Figure 7. A single broad peak centered on 7.8 ppm was present for Na-FBAG. A very weak shoulder was present at 3 ppm, whereas two peaks were present at 5.5 ppm and 2.8 ppm for K-FBAG. The peak at 5.5 ppm was relatively broad, whilst the peak at 2.8 ppm was much sharper.

**Figure 8:**
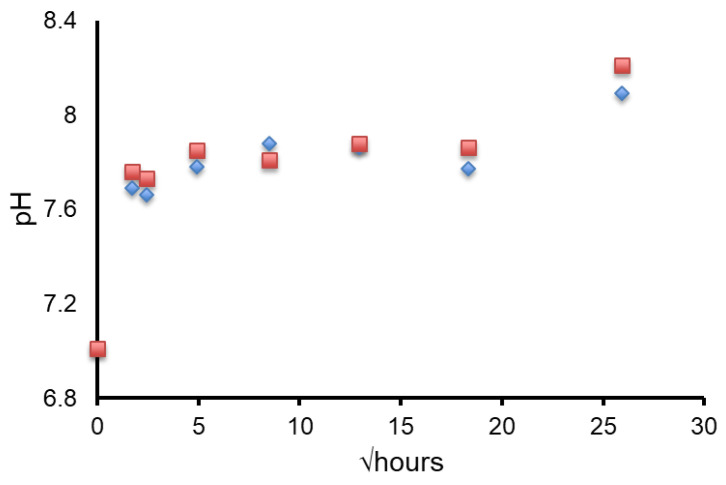
pH changes for Na-FBAG (♦) and K-FBAG (■) after immersion in AS7 for up to 4 weeks. Errors smaller than data points.

**Figure 9:**
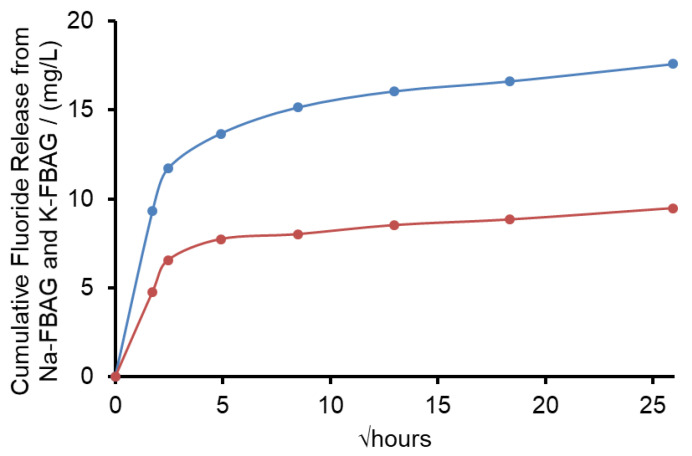
F^−^ Ion concentrations for Na-FBAG (●) and K-FBAG (●) after immersion in AS7 for up to 4 weeks. Errors comparable to data points.

**Figure 10:**
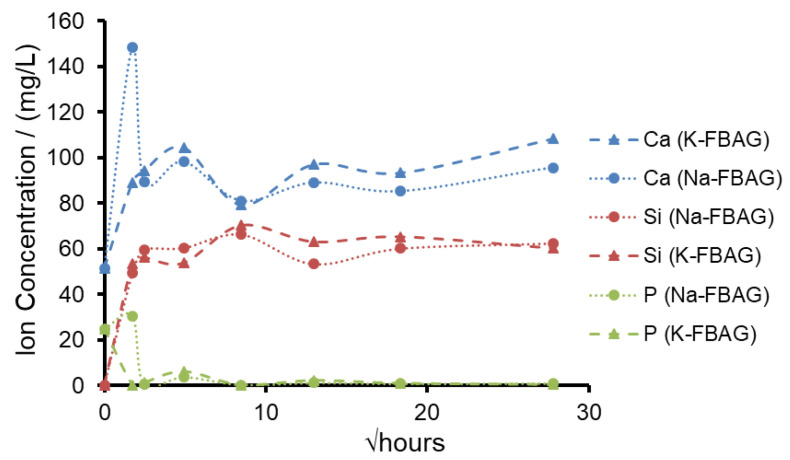
Concentration of Ca (blue), Si (red) and P (green) after immersion in AS7 Na-FBAG (circle) and K-FBAG (triangle) for up to 4 weeks.

**Figure 11:**
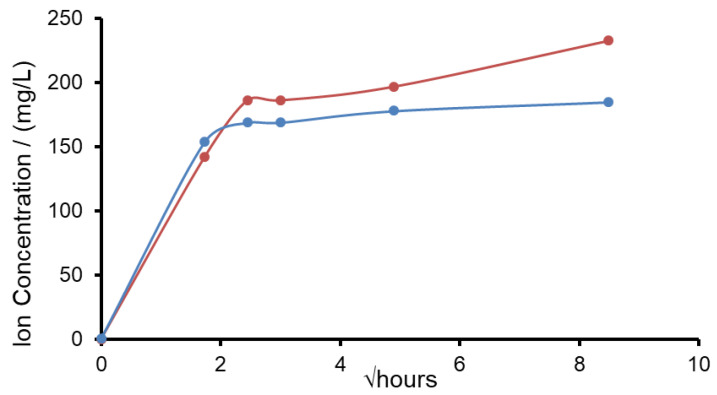
Release of sodium from Na-FBAG (●) and potassium ions from K-FBAG (●) in TB for up to 3 days.

The XRD patterns after immersion in AS7 are shown in Figure 1 and Figure 2. Both glasses formed apatite before 3 h as evidenced by diffraction lines at 25.8 and 31–33° two theta. In addition, small amounts of crystalline calcite with a diffraction line at 29.4° two theta were also found. The ATR-FTIR spectra are shown in Figure 3 and Figure 4. The non-bridging oxygen vibrations at 930 cm^−1^ were lost and sharp peaks at 1032 cm^−1^, 600 cm^−1^ and 560 cm^−1^ corresponding to the v_3_, v_4_ and v_5_ P-O vibrations in apatite respectively [30]. The Na-FBAG and K-FBAG both show a sharp peak in their ^31^P MAS-NMR spectra at 2.7 ppm after immersion in AS7. They both show a sharp peak in their ^19^F MAS-NMR spectra at approximately −103 ppm after immersion. There was a weak shoulder at about −85 ppm with both glasses that corresponded to the substitution of carbonate (CO_3_^2−^) for phosphate (PO_4_^3−^) in the apatite lattice with an associated fluoride ion. The pH change of the AS7 after immersion for Na-FBAG and K-FBAG are shown in Figure 8. The pH increased upon immersion from a starting pH of 7 to a pH of about 7.8 after 3 h and remained approximately constant thereafter. There was no significant difference between the two glasses in terms of their pH rising capability. The concentration of F^−^ in solution was highest for both glasses after immersion for 3 h but was lower for K-FBAG than for Na-FBAG (Figure 9). After 3 h very similar F^−^ profiles were found for both glasses in solution, but the F^−^ was lower for K-FBAG. The data for the Ca, Si and P after immersion in AS7 are given in Figure 10. The silicon and phosphorus ion release data upon immersion into TB are presented in Figure 11.

## 4. Discussion

The fact that both glasses gave no sharp diffraction lines by XRD would indicate that both glasses are amorphous. However, the weak peaks at 560 and 600 cm^−1^ in the ATR-FTIR spectrum of K-FBAG were consistent with a small amount of apatite having formed [24]. The sharp peak in the ^31^P MAS-NMR spectrum of K-FBAG at 2.8 ppm also corresponds to that of apatite, whilst the peak at 5.5 ppm corresponded to an amorphous phosphate. The broad peak in the ^31^P spectrum for Na-FBAG corresponds to a mixed calcium sodium orthophosphate. Previous studies have shown the chemical shift is directly proportional to the (CaO + CaF_2_)/(CaO + CaF_2_ + Na_2_O) [24,31,32] The calculated value for Na-FBAG based on this assumption was 7.6 ppm, which agreed very well with the experimental value of 7.8 ppm. The reason the chemical shift moved linearly with the Ca:Na ratio was that Ca^2+^ and Na^+^ had similar charge to size ratios. The K^+^ ion had a much lower charge to size ratio than Ca^2+^ and therefore PO_4_^3−^ and F^−^ ions might have a stronger preference for Ca^2+^ ions than for K^+^ ions. There is very limited data for potassium containing bioactive glasses in the literature but based on the chemical shifts for K_3_PO_4_ [33] and a calcium orthophosphate we would expect a chemical shift of 8.3 ppm for the K-FBAG glass. The actual chemical shift was much lower than this at 5.5 ppm. The multiple peaks in the ^19^F spectrum for Na-FBAG correspond to mixed F-Ca/Na(n) sites [18]. In contrast, there was no evidence of mixed F-Ca/K(n) sites in the K-FBAG glass.

The data taken together would indicate that both the orthophosphate and the F^−^ ions have a much stronger affinity for Ca^2+^ cations than for K^+^ ions, whilst they have no preference between Ca^2+^ and Na^+^ ions. This probably reflects the greater differences in charge to size ratio between K^+^ and Ca^2+^ than between Na^+^ and Ca^2+^. K-FBAG has undergone possible nanocrystallization to FAp, which is probably favored by the stronger association of both PO_4_^3−^ and F^−^ ions with Ca^2+^ cations. The crystallite size must be << 50 nm to give the amorphous XRD pattern. Some slight support for this comes from the study by Angelopoulou et al.’s [32] study where Na_2_O was replaced by K_2_O in the 45S5 bioglass composition where they observed a weak shoulder at 3 ppm corresponding to calcium orthophosphate. The potassium ions must be associated more with non-bridging oxygens (NBOs) attached to the silicons in the K-FBAG glass.

The DSC trace for Na-FBAG exhibited a glass transition (T_g_) of 520 °C whilst that for K-FBAG was somewhat higher at 550 °C (Figure 5). This reflected the fact that some of the fluorine was present as nanocrystalline FAp, which reduced the fluorine content of the residual glass phase, thereby increasing the T_g_. The crystallization temperature (T_c_) was slightly higher for Na-FBAG than K-FBAG at 676 °C and 665 °C respectively.

The particle size analysis for the two glasses were not significantly different from one another. Furthermore, for dentinal tubule occlusion both glasses had a significant fraction of glass particles smaller than 4 microns and were therefore small enough to go into the tubules to cause tubule occlusion.

The fluoride concentration observed for K-FBAG was lower than that for Na-FBAG at the earliest immersion time of 3 h and this reflects again the fact that some of the F^−^ ions were present in nano FAp crystals within the glass. The F^−^ ions within the nano FAp crystals present in K-FBAG will not be available for release, which explains the lower cumulative F^−^ ion concentration in solution.

The pH change upon immersion of both Na-FBAG and K-FBAG were both the result of ion exchange of Na/K^+^ and Ca^2+^ ions for H^+^ ions in the AS7. This ion exchange process reduced the H^+^ ion concentration in the AS7 resulting in a pH increase. The two glasses had almost identical pH rises as a function of time. This reflects the fact that the ion exchange occurred with the silicate part of the glass structure, which was very similar between the two glasses.

The ATR-FTIR spectra show the loss of the NBO vibrations upon immersion at 880 and 920 cm^−1^ and the formation of strong bands at 560, 600 and at 1030 cm^−1^, corresponding to a crystalline calcium orthophosphate of which apatite is a possibility. In addition, there were weaker bands at 870, 1420 and 1480 cm^−1^ corresponding to carbonate vibrations suggesting a carbonated apatite was forming. XRD following immersion shows the formation of an apatite PDF 09-0432 with the 002 diffraction line at approximately 25.8° two theta and a broad peak at 30–34° two theta corresponding to the overlapping 211, 112, 300 and 202 diffraction lines of apatite. In addition, a weak diffraction line at 29.4° two theta was present corresponding to calcite (CaCO_3_) that indicates that not all the CO_3_^2−^ ions were within the apatite lattice. The formation of apatite was apparent by 3 h by both ATR-FTIR and XRD. 

Figure 10 shows the concentrations of Ca, P and Si measured by ICP-OES. The release of silicon for the two glasses was almost identical, which was as expected, as the two glasses had the same NC of 2.16 and would be expected to degrade at very similar rates. The Si concentration in AS7 saturated at about 60 mg/L before 6 h, corresponding to the solubility limit for Si. The P declined upon immersion of both glasses from the initial value of 31 mg/L to very low values. This was consistent with the consumption of P in the formation of FAp. Since both glasses were deficient in phosphate compared to the apatite stoichiometry, very little phosphorus was detected in solution.

The calcium concentrations in solution were similar for both glasses except at 3 h where there were higher Ca and P concentrations in solution for Na-FBAG compared to K-FBAG, which may be related to the mixed Ca/Na orthophosphate observed in the Na-FBAG glass being more soluble than the Ca orthophosphate in the K-FBAG.

Sodium azide was added as a bactericide to AS7 and potassium phosphate salts were present in the AS7 composition, so the sodium and potassium concentrations released from the glass could not be measured accurately. For this reason, the release of potassium and sodium from both glasses was investigated after immersion in TB at pH 7. The results are shown in Figure 11. The release profiles for potassium from the potassium glass was very similar to that sodium glass. After 9 h of immersion the potassium glass had released 61% of its potassium ions, whilst the sodium glass had released 66% of its sodium ions. Typically, potassium nitrate-based toothpastes contained 3% KNO_3_ by weight equivalent to 1.16 weight percent potassium. The K-FBAG glass contained just over 25% by weight potassium. If the glass was added to the toothpaste at 5% by weight, the K content would be 1.25% by weight comparable to a potassium nitrate toothpaste. Much like we have salivary fluoride clearance in the mouth for soluble fluoride we are going to get salivary potassium clearance of potassium concentrations above normal salivary levels. The ability of the glass particles to slowly deliver potassium ions is a very attractive feature, particularly if the glass particles can enter the dentinal tubules and the potassium ions released can travel down the tubules to deactivate the nerves in the pulp chamber.

## 5. Conclusions

Potassium can be substituted for sodium in fluoride bioglasses with very little change in the dissolution rate, pH change and the rate of formation of FAp. There are upon substitution some distinct changes in the structure of the potassium containing glass, including the absence of mixed F-Ca/K(n) species that leads to a slight nanoscale crystallization to FAp and this serves to reduce slightly the available fluoride for release.

## Figures and Tables

**Table 1:** Composition of 45S5 (NovaMin and BioMinF).

Glass	SiO_2_	CaO	Na_2_O	P_2_O5	CaF_2_	NC
45S5	46–48	25–27	22–25	2.4–2.6	0	2.16
BioMinF (Na-FBAG)	36–40	28–30	22–24	4.0–6.0	1.5–3.0	2.08

**Table 2:** Composition of 45S5 (NovaMin and BioMinF).

Glass	D10 (μm)	D50 (μm)	D90 (μm)
Na-FBAG	4.17	16.2	37.2
K-FBAG	3.58	12.3	29.9

## Data Availability

Not applicable.

## Sample Availability

Samples of the compounds are available from the authors.
